# Effectiveness, safety and cost-effectiveness of vaporized nicotine products versus nicotine replacement therapy for tobacco smoking cessation in a low-socioeconomic status Australian population: a study protocol for a randomized controlled trial

**DOI:** 10.1186/s13063-022-06644-8

**Published:** 2022-09-14

**Authors:** Bridget C Howard, Hayden McRobbie, Dennis Petrie, Daniel Barker, Colin Mendelsohn, Jack Anderson, Ron Borland, Felix Naughton, Piotr Tutka, Nick Zwar, Veronica C Boland, Alexandra Aiken, Anthony Shakeshaft, Coral Gartner, Robyn L Richmond, Wayne Hall, Richard P Mattick, Michael Farrell, Ryan J Courtney

**Affiliations:** 1 National Drug and Alcohol Research Centre, University of New South Wales, R1 Building, 22-32 King St, Randwick, Sydney, NSW Australia; 2 Centre for Health Economics, Monash Business School, Monash University, Caufield, Australia; 3 Hunter Medical Research Institute, New Lambton Heights, Australia; 4 School of Medicine and Public Health, University of Newcastle, Newcastle, Australia; 5 Sydney, Australia; 6 School of Psychological Sciences, University of Melbourne, Melbourne, Australia; 7 Behavioural and Implementation Science Group, School of Health Sciences, University of East Anglia, Norwich, UK; 8 Department of Experimental and Clinical Pharmacology, University of Rzeszow, Rzeszow, Poland; 9 Faculty of Health Sciences and Medicine, Bond University, Gold Coast, Australia; 10 School of Population Health, Faculty of Medicine and Health, University of New South Wales, Sydney, Australia; 11 School of Public Health, Faculty of Medicine, The University of Queensland, Brisbane, Australia; 12 The National Centre for Youth Substance Use Research, The University of Queensland, Brisbane, Australia

**Keywords:** Cost-effectiveness, Electronic cigarettes, Randomized controlled trial, Smoking cessation, Social disadvantage, Tobacco

## Abstract

**Background:**

In Australia, tobacco smoking rates have declined but inequalities remain with significantly higher smoking prevalence among low-socioeconomic populations. Clinical trial data suggest vaporized nicotine products (VNPs) aid smoking cessation. Most VNP trials have used refillable tank systems, but newer generation (pod) devices now comprise the largest market share yet have limited clinical trial evidence on safety and effectiveness. This study evaluates the effectiveness, safety and cost-effectiveness of VNPs (pod and tank device) compared with nicotine replacement therapy ([NRT]—gum or lozenge) for smoking cessation.

**Methods:**

This is a two-arm, open-label, superiority, parallel group, randomized controlled trial (RCT) with allocation concealment and blinded outcome assessment. The RCT is conducted at the National Drug and Alcohol Research Centre at the University of New South Wales, Sydney, Australia. Participants are people who smoke daily, are interested in quitting and receive a government pension or allowance (*N* = 1058). Participants will be randomized (1:1 ratio) to receive 8 weeks of free: VNPs, with pod (40 mg/mL nicotine salt) and tank device (18 mg/mL freebase nicotine) in mixed flavours; or NRT (gum or lozenge; 4 mg). All participants will receive daily text message behavioural support for 5 weeks. Assessments will be undertaken by telephone at baseline, with three follow-up calls (two check-in calls within the first month and final follow-up at 7 months post randomization) to ascertain smoking status, treatment adherence and adverse events. The primary outcome is 6-month continuous abstinence verified by carbon monoxide breath test of ≤5ppm at 7-month follow-up. Safety and cost-effectiveness of VNPs versus NRT will also be evaluated.

**Discussion:**

Further data are required to strengthen certainty of evidence for VNPs aiding smoking cessation, particularly for newer generation pod devices. To our knowledge, this trial is the first to offer choice of VNPs and no comparative effectiveness trial data exists for new pod devices. If effective, the findings can inform wider implementation of VNPs to aid smoking cessation in a priority group.

**Trial registration:**

Australian New Zealand Clinical Trials Registry ACTRN12621000076875. Registered on 29 January 2021. https://www.anzctr.org.au

**Supplementary Information:**

The online version contains supplementary material available at 10.1186/s13063-022-06644-8.

## Background

In Australia, 10.7% of Australians (aged 18 or older) smoke daily (approximately 2.1 million people) [1]. Smoking rates are higher among low-socioeconomic (low-SES) groups in Australia and globally [1–3]. A recent (2020–2021) national survey of Australians reported that persons from disadvantaged areas were *more than three times as likely* to be current daily smokers than those from most advantaged areas, 17.8% vs. 5.8%, highlighting this important driver of health inequalities [1]. Low-SES smokers face multiple barriers to smoking cessation including financial stress, high smoking rates in their social network and higher nicotine dependence [4, 5]. Despite public health efforts, there is a lack of evidence on effective smoking cessation interventions for low-SES groups [6].

In most countries, the use of vaporized nicotine products (VNPs), commonly referred to as electronic cigarettes or vaping, has increased over the last decade. Recent (2019) Australian National Drug Strategy Household Survey found that 39% of adults who smoke had ever used a VNP, compared with 31% in 2016 [3]. Furthermore, 32% of people who use VNPs report using them to quit smoking, 22% to reduce the number of cigarettes smoked and 17.8% to stop them going back to smoking regular cigarettes [3]. A national survey in the United Kingdom (UK) found 33% of smokers report using VNPs for smoking cessation, compared with 22% who report using nicotine replacement therapy (NRT) [7].

VNPs have the potential to be more effective than NRT because of their superior nicotine delivery [8–13], a greater reduction in reported urges to smoke [10, 14] and greater sensorimotor replacement, and they appear to have greater adherence than NRT [10, 11, 15]. Furthermore, VNPs are generally cheaper than cigarettes and so are considered more affordable for low-income populations. In recent years, newer fourth-generation pod VNP devices have emerged, which appear to have improved pharmacokinetic data compared to early-generation devices [11, 16, 17]. Fourth-generation VNPs can produce concentrations of nicotine comparable to NRT [8], with faster nicotine delivery and a pharmacokinetic profile similar to smoking [12, 13, 18, 19]. Regarding safety, clinical trial data on VNPs report a range of relatively minor short-term side effects such as headache, dry mouth, cough, nausea and mouth and throat irritation [20]. Whilst VNPs are generally considered much less harmful than cigarette smoking because they expose users to fewer harmful and potentially harmful chemicals [9], data on adverse effects associated with long-term VNP use are required.

Clinical trial evidence for VNPs aiding smoking cessation is limited by the number and quality of trials. A 2021 Cochrane review of 61 studies, including 34 randomized controlled trials (RCTs) [20], found moderate-certainty evidence (4 studies, 1924 participants) that VNPs were more effective than NRT for long-term cessation rates (risk ratio (RR) 1.53, 95% confidence interval (CI) 1.21 to 2.93; *I*^2^ = 0%). A longstanding limitation of these trials, documented within this review, is that most RCTs are small-scale RCTs, and some of the larger trials were underpowered to detect statistically significant differences between VNPs and comparators [20]. Limitations of previous trials include poor nicotine delivery or low concentrations of nicotine in early-generation VNPs and poor to modest retention of participants at final follow-up [20]. To our knowledge, there is no existing comparative effectiveness clinical trial data on fourth-generation pod VNPs for smoking cessation.

Given Australia currently bans the sale of nicotine liquids for vaping without a prescription, whereas these products are far more accessible in the UK, United States (US) and New Zealand, there is a need for more Australian data comparing VNPs with standard smoking cessation medications, such as NRT. The main objective of this trial is to evaluate the effectiveness, cost-effectiveness and safety of VNPs compared with oral NRT. Given the significantly higher smoking rates among low-SES populations, this trial specifically evaluates the comparative effectiveness of VNPs to NRT in this group. Pragmatic use of nicotine products for smoking cessation involves limited behavioural support and choice from a range of products and flavours. In this trial, to partially mimic real-world settings, we offer participants a choice of VNP or NRT product in each arm respectively and provide minimal behavioural support via text messages [21].

### Aim

To evaluate the effectiveness, safety and cost-effectiveness of VNPs (pod device with 40 mg/mL nicotine salt and tank device with 18 mg/mL freebase nicotine) plus text message behavioural support compared with oral NRT (gum or lozenge; 4 mg) plus text message behavioural support for smoking cessation in Australian low-SES daily smokers who are willing to quit.

### Hypotheses

It is hypothesized (H), compared to comparator group (NRT), the intervention group (VNP) will show at 7-month follow-up:

- H1 (effectiveness): a significantly higher 6-month biochemically verified continuous abstinence rate;
- H2: (safety): comparable short-term safety outcomes (demonstrated via proportion of serious adverse events (SAEs) and adverse events (AEs) reported for NRT and VNP arms); and
- H3 (cost-effectiveness): greater cost-effectiveness in achieving 6-month continuous abstinence.

## Methods

### Design

This is a parallel two-group, superiority, open-label RCT with a 1:1 treatment allocation ratio. This study is reported using the SPIRIT reporting guidelines [22].

### Setting

The trial will be undertaken in New South Wales (NSW), Australia. Screening and consenting procedures will be completed at the Trial Coordinating Centre (TCC) at the National Drug and Alcohol Research Centre, University of New South Wales, Sydney, Australia. Baseline and follow-up computer-assisted telephone interviews (CATIs) will be completed by an independent contract research organization (CRO)—the Social Research Centre located in Melbourne, Australia.

### Participants

Participants will be people who receive a government pension or allowance (a proxy of low-SES) who smoke daily from Sydney and the wider catchment region, NSW.

#### Inclusion criteria

Participants will be included if they are at least 18 years of age; in receipt of a government pension or allowance; current daily smoker; interested in quitting smoking and willing to use the medication provided (VNP or NRT); agree to use the allocated study product and refrain from using other quit smoking medications whilst using the study products; speak English; able to provide informed consent; have a mobile telephone that can receive text messages; willing to allow the research team and study physician to access data for quality assurance and maintain integrity of trial data for the purposes of (i) the study physician to make contact if required; (ii) the CRO to conduct two CATIs; and (iii) mailing out of study products; available for follow-up over a 7-month period; and willing to complete baseline and follow-up CATIs.

#### Exclusion criteria

Exclusion criteria comprise the following: currently participating in another quit smoking programme/study; currently using any quit smoking medications or products; diagnosed with unstable angina; hospitalized for stroke, heart attack or another heart-related condition in the last 2 weeks; women who are pregnant, breastfeeding or planning to become pregnant in the next 7 months; or deemed medically unfit, by the study physician, to participate at the time of screening.

### Recruitment

Participants from Sydney and the wider catchment region are recruited predominately through study advertisements across online and social media platforms such as Facebook advertisements. Participants in a recently completed clinical trial comparing cytisine versus varenicline for smoking cessation who consented to being contacted about future research and were receiving a government pension were also invited to take part [23].

Advertising briefly informs prospective participants about the research study and advises that participation will involve nicotine replacement products and support to help them quit (at no financial cost). Advertisements include a link to the study webpage, where people can view the Participant Information Statement, complete the contact form to receive a call-out from TCC staff to complete screening, or contact the TCC staff directly via the toll-free study number or email. Participants can complete the consent form online or as a hard-copy version sent via post. All participants will be provided with a Participant Information Statement and consent form for their records, either via mail or email, after completing the screening and consent call.

After consent is obtained, participants are referred to the study physician who will review screening files and provide final approval for study enrolment. The study physician will evaluate participants with a self-reported cautionary condition via tele/video conferencing before progressing the participant to baseline data collection and randomization. The inclusion of participants with any cautionary condition will be at the study physician’s discretion, after the potential benefits have been weighed against the possible risks. Further details on cautionary conditions and the screening process can be found in the Supplementary Materials (Additional File 1).

### Randomization: allocation concealment and sequence generation

**Fig. 1:**
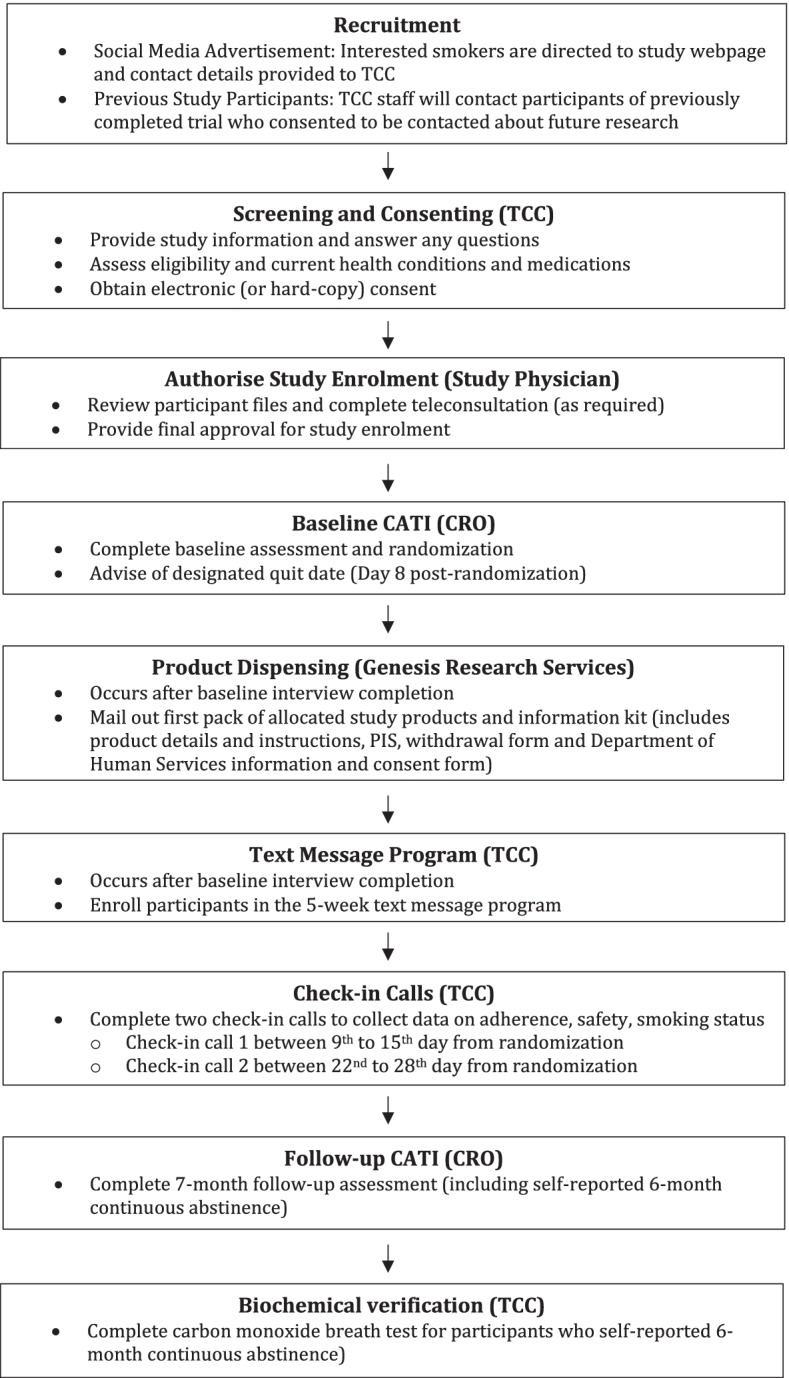
Study flow diagram

Following study physician sign off, participants will be referred to the CRO for the baseline CATI and randomization. Further information is provided in the study flow diagram (see Fig. 1). Participants will be randomized to a treatment group after completion of the baseline CATI. The data collection system at the CRO (UNICOM® Intelligence) will assign a unique randomization number to each participant using a pre-generated randomization list embedded in the system. Allocations will be undertaken using the permuted block design (with unequal block sizes of 12 and 16). After baseline interview completion, the system randomizes participants to treatment arms in a 1:1 ratio. Only the independent statistician and script programmers will have access to the pre-generated randomization list.

### Blinding

As there is a difference in dosage form and regimen, participants and research staff who perform day-to-day activities (including distributing products and conducting check-in calls) cannot be blinded. This means that only single blinding is possible (i.e. only the independent outcome assessor at the independent CRO will be blind). The interviewers at the CRO who conduct outcome assessments will use the unique study ID (i.e. randomization number) and will not know the participant’s treatment allocation.

### Study treatments

Participants will be randomly allocated to either VNP (pod device and tank device with nicotine e-liquids) or NRT (a choice of nicotine gum or lozenge). After randomization, the CRO will inform each participant about their allocation and provide details on use of the study products and the designated quit day (i.e. day 8 post randomization). Participants will receive, alongside standard product monograph, a guide on how to use the study product, as well as a consent form for releasing their Medicare (prescription drugs and federally subsidized out of hospital health services) claims information for the cost-effectiveness analysis, although Medicare consent is not a requirement for study participation. Participants will be advised to follow the dosing regimen as recommended by the manufacturer for NRT and use VNPs ad libitum. The study treatments are provided in two deliveries of 4 weeks’ supply and are dispatched from either the TCC or Genesis Research Services, Broadmeadow, NSW, Australia. Participants who report not using their allocated product in their second check-in call or do not complete their second check-in call will not be sent the second pack; these participants are advised to contact the TCC if they wish to receive their second pack.

#### Intervention group

Participants in the VNP arm will receive up to 8 weeks’ supply of nicotine e-liquid to be used in a personal vaporizing device. Each participant receives two devices: a tank device (Innokin Endura T18) with 18 mg/mL freebase nicotine e-liquid and a pod device (alt.) with 40 mg/mL nicotine salt liquid in prefilled pods. Each type of nicotine e-liquid is provided in three flavours (tobacco, menthol and fruit). This study allows for participant preferences (device type and flavours) to be accommodated in their second pack of VNP products.

#### Comparator group

Participants in the NRT arm receive up to 8 weeks’ supply of 4 mg nicotine gum or lozenges. Participants choose their preferred NRT, gum or lozenges, in mint flavour, and are supplied with 15 pieces of gum or lozenges per day in two deliveries of 4 weeks’ supply.

### Study treatments (cont.)

Participants in both groups can change their treatment preference for their second pack delivery. For example, participants can elect to change oral NRT form or elect to use e-liquids for a single VNP device or both devices provided. Participants in the VNP arm can also elect for single or mixed flavours in their second pack. Further details on the flexible treatment approach are provided in the Supplementary Materials (Additional File 2).

Participants in both arms are provided information on how to access their assigned product to ensure they are able to continue treatment if required beyond the 8-week treatment period. Participants in the NRT arm will be advised they can purchase their products over the counter from a community pharmacy/chemist or from supermarkets. Participants in the VNP arm will be provided with a prescription from the study physician for nicotine e-liquid and advised that they can purchase and import up to 3 months’ supply per order in accordance with the Australian Therapeutic Goods Administration Personal Importation Scheme or purchase from a local pharmacy. This ensures that both arms have similar access to products after the treatment period.

#### Text message quit support

All participants will receive behavioural support via text message. Mobile phone-based quit smoking support is effective [21, 24] and is recommended by the World Health Organization (WHO) [25]. As a base platform, text messages were developed from the WHO Be He@lthy, Be Mobile Handbook on how to implement mTobacco Cessation [25] with wider consultation obtained from members of the Trial Steering Committee to guide development of modified, or newly developed general, VNP-specific or NRT-specific text messages. Text messages were also developed specifically for the purposes of the trial. The text message support programme is provided for a period of 5 weeks (35 days) and comprises a total 112 text messages, including 93 general messages and 19 treatment-specific messages. The text message programme includes information on how to use the study products; tips for coping with nicotine withdrawal symptoms and side effects of their VNP/NRT products; study progress updates; goals and planning; relapse prevention and motivational ‘feel good’ messages.

### Concomitant care

Concomitant medications are recorded at screening and check-in calls.

## Data collection and measurements

### Follow-up

The independent CRO will complete all assessments at baseline and follow-up (7 months post randomization) using a structured CATI programme. Participants will be reimbursed $40 for competing their 7-month telephone interview.

### Check-in calls

All participants will receive two check-in calls. Non-adherence and AEs usually occur during the initial phase of treatment, and hence two check-in calls will be completed during the first month of treatment. Data on smoking abstinence will be collected and researchers provide brief behavioural support based on smoking status. These calls will be completed by trained unblinded research staff at the TCC.

### Treatment adherence

Participants will receive clear instructions on the dosage regimen immediately after randomization by the CRO. Participants will also receive consumer medicine information and a leaflet detailing dosage regimen, designated quit day, common side effects and study contact details along with the medication pack. Treatment adherence will be monitored during the check-in calls, and those who are not adherent will be encouraged to use the medication as per the schedule. Self-reported treatment adherence will also be assessed at 7-month follow-up. Participants can choose to discontinue treatment at any time, and participants will be advised to discontinue treatment if any of the following occurs: (i) an SAE, concurrent illness or medical condition that the Principal Investigator and study physician deem continued treatment would not be in the participant’s best interest, (ii) pregnancy or (iii) study termination.

### Measurements

The measures, and timepoints at which these are collected, are summarized in Table 1.

**Table 1:** Measures

	Study period				
	Screening	Baseline (day 1)	Check-in call 1 (days 9–15)	Check-in call 2 (days 22–28)	Final follow-up (7 months post randomization)
Enrolment					
Eligibility screen	X				
Informed consent	X				
Treatment allocation (randomization)		X			
Assessments					
Demographic information: age, sex, postcode, receipt of government pension	X				
Sociodemographic information: ancestry, education, marital status, family composition		X			
Socioeconomic demographics: employment status, income		X			X
Study treatment adherence			X	X	X
Adverse events			X	X	X
Self-reported abstinence			X	X	X
Smoking information: daily consumption, withdrawal symptoms, urges [26]		X	X	X	X
Smoking (and quitting) history, self-efficacy, attitude, identity [27–30]		X			
Financial stress [31, 32]		X			X
Alcohol use (AUDIT-C) [33]		X	X	X	
Cannabis use and drug/alcohol treatment		X			
Psychological distress (K10) [34]		X			
Quality of Life (EQ5D-5L) [35]		X			X
Respiratory symptoms [36, 37]		X	X	X	X
Study treatment feedback					X
Acceptability of text message support					X
Concomitant medications	X		X	X	
Carbon monoxide verified abstinence					X

#### Primary outcome

The primary outcome will be self-reported 6-month continuous abstinence with biochemical verification of abstinence at the 7-month follow-up. Continuous 6-month abstinence will be defined as having remained abstinent for 6 months (self-report of having smoked no more than five tobacco cigarettes in that time), and a carbon monoxide (CO) level of ≤5 ppm to verify abstinence [38]. Only participants self-reporting continuous abstinence from smoking tobacco at final follow-up will be biochemically verified. CO level in exhaled air will be measured using a hand-held Smokerlyzer device (Micro^+^ or iCO^TM^). A participant with an exhaled CO level of ≤5 ppm will be considered abstinent [39]. Participants will be asked to either visit the TCC, have a trained researcher attend their home to perform this test or complete a mailed hand-held iCO™ CO breath test remotely with accompanying instructions. Participants who opt to complete remote iCO™ Smokerlyzer® will be mailed this device and instructions and complete the test on a video call with trained research staff from the TCC. Participants will be reimbursed $40 for their time. Participants who self-report abstinence but do not complete the CO breath test will be classified as smoking for the primary outcome.

#### Secondary outcomes

Secondary outcomes are:

(i) Self-reported continuous abstinence at 7-month follow-up (self-report of smoking not more than five cigarettes from the final follow-up).
(ii) Self-reported 7-day point prevalence abstinence at check-in calls and 7-month follow-up (self-report of not having smoked at all [not even a puff]).
(iii) Reduction in number of cigarettes smoked per day from baseline to 7-month follow-up; measured via cigarette consumption among those not abstinent from smoking (number of cigarettes smoked per day; mean reduction and proportion of participants that achieved ≥ 50% reduction at follow-up compared to baseline cigarette consumption).

### Safety monitoring

Participants will receive two check-in calls during the first 4 weeks of the 8-week treatment period. The check-in calls assess smoking status, treatment adherence, AEs or SAEs, and any changes to concomitant medication. A toll-free number is available for participants to self-report the occurrence of any AEs and seek advice from the TCC staff. A Clinical Advisory Committee, TCC research team and an independent Data Safety Monitoring Committee (DSMC) will oversee participants’ safety, medication use and safety data collection. Any AE or SAE will be followed up until resolved or insufficient follow-up is established, this can extend beyond the final 7-month follow-up.

An AE will include any illness, signs or symptoms or clinically significant abnormality that has appeared or worsened during the clinical trial, regardless of its causal relationship to the study medications. All AEs will be carefully evaluated and summarized using Medical Dictionary for Regulatory Activities (MedDRA) coding. The severity will be assessed using Common Terminology Criteria for Adverse Events version 4.0 general guidelines (i.e. grade 1: mild AE; grade 2: moderate AE; grade 3: severe AE; grade 4: life-threatening or disabling AE; and grade 5: death related to AE). Causality will be assessed using the WHO criteria for causality assessment (certain, probable/likely, possible, unlikely, conditional/unclassified and unassessable/unclassifiable).

There are no formal stopping rules set for this study, but an independent DSMC will evaluate the safety data emerging from the study at least biannually and make recommendations on whether to continue the trial without changes, continue with changes or terminate the trial.

### Sample size

The study plans to enrol 1058 participants in total (529 per group) to provide a power of 80%, for a two-sided significance level of 0.05 and assuming a 20% loss to follow-up. In Hajek et al. [40], 12-month verified abstinence rates in the NRT group were 9.9%. Assuming ~25% relapse between 6 and 12 months [41], this would translate to a 6-month abstinence rate of 13.2%. However, we expect abstinence rates to be 40% lower in the comparator arm in this trial as we are (a) using lower intensity behavioural support (text messaging compared to multi-session face-to-face), (b) providing a single NRT (the majority of participants in Hajek et al 2019 used combination treatment) and (c) recruiting participants from a population that is expected to have low quit self-efficacy. Using these assumptions, the estimated verified 6-month continuous abstinence rate in our NRT group is 8%. Using the risk ratio of 1.8 found in the Hajek et al. study, we assume a 6-month verified continuous abstinence rate of 14% in our VNP group. In a recent smoking cessation RCT among Australian low-SES smokers (*n* = 1047), 84% retention was achieved at final 8-month follow-up, despite higher study demands on participants (length and number of calls) [42]. Given the current study has fewer study demands on participants and a shorter follow-up period compared to the previous study, we expect the loss to follow-up to be at least comparable.

### Data management

The data captured at the TCC will use a specifically designed database which has a secure gateway (GlobalProtect). The study physician and product dispensing research team will have restricted access to the database. The data collected at the CRO will be stored in an electronic database, UNICOM® Intelligence. The secured file exchange portal, ShareFile®, will be used for transferring files between the TCC and CRO databases.

### Data analysis

#### Baseline characteristics

Baseline characteristics of the NRT and VNP groups will be presented using frequency and percentages for categorical variables and means/standard deviations or medians/interquartile ranges for continuous measures.

#### Treatment effects

A Bayesian beta-binomial posterior distribution for the quit proportions will be constructed for the NRT and VNP group separately and one million random draws from each posterior distribution will be taken. Superiority of VNP over NRT will be established if the posterior probability of quitting in the VNP arm is greater than corresponding posterior probability in the NRT arm in 97.5% of random draws. A beta(1,1) prior (non-informative) will be used for the primary analysis, but sensitivity analyses will use informative beta priors based on previously published results for NRT and VNP. Given the field is evolving rapidly with new studies, it is inappropriate to pre-specify exact priors at this stage. The trial statistical analysis plan will be updated prior to data analysis and the most updated priors will be selected from the latest Cochrane review. A similar Bayesian analysis will be conducted for the proportions of SAEs and AEs in each study arm, and 95% credible intervals will be reported for abstinence rates in each arm and for the difference in quit proportions.

The primary effectiveness analysis will be conducted in accordance with the intention-to-treat principle. All randomized participants will be included in the analysis set and will be classified as still smoking unless self-reported continuous abstinence is verified by CO breath test. The primary analysis will consider individuals with missing smoking status at follow-up as treatment failures. Sensitivity analyses will be conducted with alternative missing data assumptions and excluding participants with protocol deviations [43]. Further details of all statistical analyses will be included in the statistical analysis plan.

#### Tolerability analysis

The primary AE outcome will be the difference in the rate of AEs between the treatment groups. The incidence of all suspected AEs will be summarized by treatment group, as follows: type, severity, causality, action taken and outcome. All randomized participants who take at least one dose of NRT or VNP will be included in the safety analyses. Comparison of the frequency of treatment withdrawal between the NRT group and VNP group will be tested using chi-square statistics. The number of participants discontinuing treatment prematurely for any reason will be summarized by treatment group and by reasons for discontinuation. The DSMC provide periodic oversight of AE data. For the primary AE outcome, the proportion of reported AEs occurring between treatment initiation and 7-month follow-up will be compared between the two treatment groups. The most frequent AEs (occurrence of ≥5%) will be presented by MedDRA term and compared between treatment groups. All SAEs will be presented by MedDRA term, event type and treatment group. An analysis of AE occurrence between VNP and NRT will also be conducted for participants reporting an AE start date within 28 days after the baseline interview. The between-group difference for the rate of AEs will be modelled using negative binomial regression. The analysis of the AEs will be summarized using the incidence rate ratio and 95% CI for the VNP group compared with the NRT group. This analysis will be two-sided and use a significance threshold of 0.05.

#### Health economic analyses

The cost-effectiveness analysis will take a healthcare perspective. Costs will be captured by staff recording time taken and resources used for assessment, administration, managing any complications or AEs, and intervention-related costs assuming that both NRT and VNP are subsidized by the government. There is a limited ability to estimate the costs of VNP if subsidized in Australia, but we will conduct sensitivity analyses under different cost scenarios. Additional cost differences relating to other general healthcare use (Medicare) will be captured through data linkage. First, a within-trial analysis will be undertaken of the additional cost per additional quitter. Given that the majority of the health benefits and cost savings from quitting are likely to be experienced post trial, the implications for quality-adjusted life years (QALYs) and future costs will be modelled over the lifetime using an individual-level simulation model which considers smoking intensity, future smoking behaviour and cessation attempts and the estimated impacts on lung cancer, chronic obstructive pulmonary disease, myocardial infarctions and stroke and the cost per QALY will be estimated. Future relapse rates, cessation attempts and other parameters for the individual-level simulation model will be estimated for Australia using the Household Income and Labour Dynamics in Australia data which includes self-reported smoking behaviour. Given the limited data available on the long-term health risk of VNP, we will model a range of plausible scenarios based on varying numbers of long-term users informed by those still using VNP at the end of follow-up and percentage reductions in smoking-related disease risk. Both the within-trial and modelled cost-effectiveness analysis will include probabilistic sensitivity analysis to explore the robustness of the conclusions and the potential implications of lower levels of adherence in non-trial setting.

## Discussion

The results of this trial will make an important contribution to the evidence base for the comparative effectiveness of VNP and NRT as smoking cessation aids. This trial is pragmatic. In the real-world setting, people who want to use VNPs for smoking cessation have a choice of products, nicotine strength and flavours. This was the case in Hajek et al., where participants were given a specific device and flavours, but were free to change, at their own expense. This study provides people with two VNP devices and mixed flavours in their first pack. Participants can tailor their second pack to their preferred device and flavours. The devices were chosen because of their popularity, safety profile and adherence to TGO 110 (Therapeutic Goods Standard for Nicotine Vaping Products Order) [44]. The exploratory aspect of this trial, flexible choice of device and flavour, will be informative on the impact of choice on treatment adherence and cessation rates.

It is important to consider the Australian regulatory context which is more restrictive than the UK, US and New Zealand environments. In Australia, people are required to have a doctor’s prescription for VNPs including nicotine concentration; however, flavours do not need to be specified. For purchase at an Australian pharmacy, doctors must be registered with the Australian Therapeutic Goods Administration as an authorized prescriber of unapproved nicotine vaping products. This trial provides participants randomized to the VNP arm with a 6-month supply prescription of nicotine e-liquid (in both tank and pod form). Whilst we provide a valid prescription, Australia’s access scheme is more complex, compared to other developed countries where they are licensed for use as a quit smoking aid, and this may impact participant’s ongoing access and treatment adherence.

Most people who make quit attempts do so without using counselling services. However, it is best practice, and a requirement of the Australian Pharmaceutical Benefits Scheme subsidization of smoking cessation medicines, to offer people behavioural support for their medication-assisted quit attempt. Based on earlier work with low-SES populations [21, 24], we opted to offer text message support. We developed the programme from a publicly available message bank (WHO) [25], which includes messages about NRT. Simple messages were created to support people using VNP, ensuring a balanced number of similar messages relevant to NRT use in the NRT arm.

Other strengths of the study are its sample size and rigorous study design. It offers flexibility for participants to complete data collection processes remotely (telephone interviews, text message behavioural support, remote breath testing), which will allow for high recruitment and retention rates and continuity during any pandemic restrictions. For numerous months of this trial, the state of NSW was in ‘COVID-19 lockdown’ but recruitment was able to continue.

This study has some limitations. Biochemical verification of smoking abstinence will be completed using CO breath testing; however, additional salivary or urinary cotinine and anabasine testing may be more reliable, albeit more costly. This study has the potential to inform policy on a quit smoking aid that could annually save millions of lives world-wide.

### Trial status

Trial recruitment commenced in March 2021 and finished in March 2022. The target sample size was met. Final data collection is expected to be completed in November 2022. Given changes to the protocol prior to recruitment, there was uncertainty as to the feasibility of remote methodological changes that were needed to ensure the conduct and implementation of a trial during the COVID-19 pandemic. The research team decided to wait until all trial procedures were acceptable and feasible (i.e. remote biochemical testing) before submitting the protocol manuscript for publication. This approach ensured full sample size was met to ensure an adequately powered trial before submitting final protocol manuscript.

## Supplementary Information

**Additional file 1. **Study Physician Process outlines the process of authorisation of participant enrolment by the study physician.

**Additional file 2. **Study Treatments details the flexible treatment approach and variations of medications provided to participants.

## Data Availability

Trial data are available on request to the Principal Investigator of the study.

## Abbreviations

AE: Adverse event
CATI: Computer-assisted telephone interview
CI: Confidence interval
CO: Carbon monoxide
CRO: Contract research organization
DSMC: Data Safety Monitoring Committee
Low SES: Low socioeconomic status
MedDRA: Medical Dictionary for Regulatory Activities
NHMRC: National Health and Medical Research Council
NRT: Nicotine replacement therapy
PIS: Participant Information Statement
QALY: Quality-adjusted life years
RCT: Randomized controlled trial
SAE: Serious adverse event
TCC: Trial Coordinating Centre
VNP: Vaporized nicotine product
WHO: World Health Organization

## References

[1] Australian Bureau of Statistics. Pandemic insights into Australian smokers, 2020-2021. https://www.abs.gov.au/articles/pandemic-insights-australian-smokers-2020-21. Accessed 4 Apr 2022.

[2] Hitchman SC, Fong GT, Zanna MP, Thrasher JF, Chung-Hall J, Siahpush M (2014). Socioeconomic status and smokers' number of smoking friends: findings from the International Tobacco Control (ITC) Four Country Survey. Drug Alcohol Depend..

[3] Australian Government, Australian Institute of Health and Welfare. National Drug Strategy Household Survey (NDSHS) 2019. https://www.aihw.gov.au/about-our-data/our-data-collections/national-drug-strategy-household-survey/2019-ndshs. Accessed 4 Apr 2022.

[4] Australian Government Department of Health and Aged Care. Smoking and disadvantage evidence brief. https://www1.health.gov.au/internet/publications/publishing.nsf/Content/smoking-disadvantage-evidence-brief~factors-smoking-levels. Accessed 29 Mar 2022.

[5] Borland R (2014). Understanding hard to maintain behaviour change: a dual process approach.

[6] Courtney RJ, Naicker S, Shakeshaft A, Clare P, Martire KA, Mattick RP (2015). Smoking cessation among low-socioeconomic status and disadvantaged population groups: a systematic review of research output. Int J Environ Res Public Health..

[7] Kock L, West R, Beard E, Kale D, Brown J. Smoking in England - trends in electronic cigarette use in England. https://smokinginengland.info/graphs/e-cigarettes-latest-trends. Accessed 29 Mar 2022.

[8] Dawkins LOC (2014). Acute electronic cigarette use: nicotine delivery and subjective effects in regular users. Psychopharmacol..

[9] Public Health England. E-cigarettes: An evidence update. https://www.gov.uk/government/publications/e-cigarettes-an-evidence-update. Accessed 29 Mar 2022.

[10] Bullen C, McRobbie H, Thornley S, Glover M, Lin R, Laugeson M (2010). Effect of an electronic nicotine delivery device (e cigarette) on desire to smoke and withdrawal, user preferences and nicotine delivery: randomised cross-over trial. Tob Control..

[11] Bullen C, Howe C, Laugesen M, McRobbie H, Parag V, Williman J (2013). Electronic cigarettes for smoking cessation: a randomised controlled trial. Lancet..

[12] Wagener T L, Floyd EL, I. S, LM. D, Frank SG, Meier E, et al. Have combustible cigarettes met their match? The nicotine delivery profiles and harmful constituent exposures of second-generation and third-generation electronic cigarette users. Tob Control. 2017;26(e1):e23-e8. 10.1136/tobaccocontrol-2016-053041 PMC5574194 27729564

[13] St Helen G, Havel C, Dempsey DA, Jacob P, Benowitz NL (2016). Nicotine delivery, retention and pharmacokinetics from various electronic cigarettes. Addiction.

[14] Adriaens K, Van Gucht D, Declerck P, Baeyens F (2014). Effectiveness of the electronic cigarette: an eight-week Flemish study with six-month follow-up on smoking reduction, craving and experienced benefits and complaints. Int J Environ Res Public Health..

[15] Steinberg MB, Zimmerman MH, Delnevo CD, Lewis MJ, Shukla P, Coups EJ (2014). E-cigarette versus nicotine inhaler: comparing the perceptions and experiences of inhaled nicotine devices. J Gen Intern Med..

[16] Caponnetto P, Campagna D, Cibella F, Morjaria JB, Caruso M, Russo C (2014). EffiCiency and Safety of an eLectronic cigAreTte (ECLAT) as tobacco cigarettes substitute: a prospective 12-month randomized control design study. PLOS One..

[17] Farsalinos KE, Spyrou A, Tsimopoulou K, Stefopoulos C, Romagna G, Voudris V (2014). Nicotine absorption from electronic cigarette use: comparison between first and new-generation devices. Sci Rep..

[18] Hajek P, Pittaccio K, Pesola F, Myers Smith K, Phillips-Waller A, Przulj D (2020). Nicotine delivery and users’ reactions to Juul compared with cigarettes and other e-cigarette products. Addiction..

[19] Hajek P, Przulj D, Phillips A, Anderson R, McRobbie H (2017). Nicotine delivery to users from cigarettes and from different types of e-cigarettes. Psychopharmacology..

[20] Hartmann-Boyce J, McRobbie H, Lindson N, Bullen C, Begh R, Theodoulou A (2021). Electronic cigarettes for smoking cessation. Cochrane Database Syst Rev..

[21] Whittaker R, McRobbie H, Bullen C, Rodgers A, Gu Y, Dobson R (2019). Mobile phone text messaging and app-based interventions for smoking cessation. Cochrane Database Syst Rev..

[22] Chan AW, Tetzlaff JM, Gøtzsche PC, Altman DG, Mann H, Berlin J (2013). SPIRIT 2013 Explanation and elaboration: guidance for protocols of clinical trials. BMJ..

[23] Courtney RJ, McRobbie H, Tutka P, Weaver NA, Petrie D, Mendelsohn CP (2021). Effect of cytisine vs varenicline on smoking cessation: a randomized clinical trial. JAMA..

[24] Boland VC, Stockings EA, Mattick RP, McRobbie H, Brown J, Courtney R (2018). The methodological quality and effectiveness of technology-based smoking cessation interventions for disadvantaged groups: a systematic review and meta-analysis. Nic Tob Res..

[25] World Health Organization (2015). Be He@lthy Be Mobile: A handbook on how to implement mTobaccoCessation.

[26] West R, Hajek P (2004). Evaluation of the mood and physical symptoms scale (MPSS) to assess cigarette withdrawal. Psychopharmacology (Berl)..

[27] Gwaltney CJ, Metrik J, Kahler CW, Shiffman Saul. Self-efficacy and smoking cessation: a meta-analysis. Psychol Addict Behav. 2009;23(1):56-66. 10.1037/a0013529 PMC3829471 19290690

[28] Dupont P, Tack V, Blecha L, Reynaud M, Benyamina A, Amirouche A (2015). Smoker's identity scale: measuring identity in tobacco dependence and its relationship with confidence in quitting. Am J Addict..

[29] Tombor I, Shahab L, Brown J, West R (2013). Positive smoker identity as a barrier to quitting smoking: findings from a national survey of smokers in England. Drug Alcohol Depend..

[30] Steinberg ML, Krejci JA, Collett K, Brandon TH, Ziedonis DM, Chen K (2007). Relationship between self-reported task persistence and history of quitting smoking, plans for quitting smoking, and current smoking status in adolescents. Addict Behav..

[31] Siahpush M, Borland R, Yong HH (2007). Sociodemographic and psychosocial correlates of smoking-induced deprivation and its effect on quitting: findings from the International Tobacco Control Policy Evaluation Survey. Tob Control..

[32] Siahpush M, Carlin JB (2006). Financial stress, smoking cessation and relapse: results from a prospective study of an Australian national sample. Addiction..

[33] Bush K, Kivlahan DR, McDonell MB, Fihn SD, Bradley KA (1998). The AUDIT alcohol consumption questions (AUDIT-C): an effective brief screening test for problem drinking. Arch Intern Med..

[34] Kessler RC, Andrews G, Colpe LJ, Hiripi E, Mroczek DK, Normand SL (2002). Short screening scales to monitor population prevalences and trends in non-specific psychological distress. Psychol Med..

[35] Herdman M, Gudex C, Lloyd A, Janssen MF, Kind P, Parkin D (2011). Development and preliminary testing of the new five-level version of EQ-5D (EQ-5D-5L). Qual Life Res..

[36] Wortel K, Van Der Werf TS, Boersma WG, Altenburg Josje, De Graaff CS (2016). Validation of a visual analogue score (LRTI-VAS) in non-CF bronchiectasis. Clin Resp J..

[37] Bestall J, Paul E, Garrod R, Garnham R, Jones P, Wedzicha J (1999). Usefulness of the Medical Research Council (MRC) dyspnoea scale as a measure of disability in patients with chronic obstructive pulmonary disease. Thorax..

[38] West R, Hajek P, Stead L, Stapleton J (2005). Outcome criteria in smoking cessation trials: proposal for a common standard. Addiction..

[39] Perkins KA, Karelitz JL, Jao NC (2012). Optimal carbon monoxide criteria to confirm 24-hr smoking abstinence. Nic Tob Res..

[40] Hajek P, Phillips-Waller A, Przulj D, Pesola F, Myers Smith K, Bisal N (2019). A randomized trial of E-cigarettes versus nicotine-replacement therapy. NEJM..

[41] Stapleton J (1998). Cigarette smoking prevalence, cessation and relapse. Stat Methods Med Res..

[42] Courtney RJ, Clare P, Boland V, Martirea KA, Bonevski B, Hall W (2017). Predictors of retention in a randomised trial of smoking cessation in low-socioeconomic status Australian smokers. Addict Behav..

[43] White IR, Horton NJ, Carpenter J, Pocock SJ. Strategy for intention to treat analysis in randomised trials with missing outcome data. BMJ. 2011;342:d40. 10.1136/bmj.d40 PMC3230114 21300711

[44] Australian Government Department of Health. Therapeutic Goods (Standard for Nicotine Vaping Products) (TGO 110) Order 2021. https://www.legislation.gov.au/Details/F2021L00595. Accessed 4 Apr 2022.

