# Supplementary material for: Effectiveness, safety and cost-effectiveness of vaporized nicotine products versus nicotine replacement therapy for tobacco smoking cessation in a low-socioeconomic status Australian population: a study protocol for a randomized controlled trial
Source: Trials. 2022 Sep 14;23:777. doi: 10.1186/s13063-022-06644-8 (PMC9473457; doi:10.1186/s13063-022-06644-8)
Supplement: Supplementary file 1 — Additional file 1. Study Physician Process outlines the process of authorisation of participant enrolment by the study physician. [file 13063_2022_6644_MOESM1_ESM.pdf]

Supplementary Material 1 – Study Physician Process

After consent is obtained, all participants are referred to the study physician who will review screening files and provide final approval for study enrolment.

Participants who at screening report:

- allergies to the active substance or any of the excipients of study products or severe allergies requiring an EpiPen;
- unstable asthma;
- a mental health condition not under control;
- use of Clozapine, Fluvoxamine, Olanzapine, Insulin, Warfarin, Aminophylline, Theophylline, Erlotinib, Riociguat, Chlorpromazine, Flecainide or Methadone and does not consent to providing their doctor's contact details to receive information about their study participation

will be marked as cautionary and will require further evaluation by the study physician prior to study enrolment. The study physician will evaluate participants with a self-reported cautionary condition via tele/video conferencing before progressing the participant to baseline data collection and randomisation. The inclusion in the trial of smokers with any cautionary condition will be at the study physician's discretion, after the potential benefits have been weighed against the possible risks.
