# Supplementary material for: Effectiveness, safety and cost-effectiveness of vaporized nicotine products versus nicotine replacement therapy for tobacco smoking cessation in a low-socioeconomic status Australian population: a study protocol for a randomized controlled trial
Source: Trials. 2022 Sep 14;23:777. doi: 10.1186/s13063-022-06644-8 (PMC9473457; doi:10.1186/s13063-022-06644-8)
Supplement: Supplementary file 2 — Additional file 2. Study Treatments details the flexible treatment approach and variations of medications provided to participants. [file 13063_2022_6644_MOESM2_ESM.pdf]

Supplementary Material 2 – Study Treatments

Study medications and materials will be delivered to participants' residential address via registered express post to ensure tracking and receipt of delivery is recorded. An initial four week supply will be mailed after baseline telephone interview completion and a second four week supply will be mailed after the second check-in call has been completed. Participants who do not complete their second check-in call or are not using the study products at the time of the call, will receive a letter in place of the study products advising them to call the study toll free number if they change their mind and wish to continue using the study products.

The second delivery of study products will contain information for both arms on accessing their respective product to ensure they are able to continue treatment if required beyond the eight-week treatment period. Participants in the NRT arm will be advised that they can purchase their products over the counter from a community pharmacy/chemist or from supermarkets. Participants in the VNP arm will be provided with a prescription from the study physician for nicotine e-liquid for both devices and advised that they can purchase and import up to 3 months' supply at a time online under the Australian Therapeutic Goods Administration (TGA) Personal Importation Scheme or purchase from a local pharmacy. This ensures that both arms have similar access to products after the treatment period.

Intervention Group

Pack 1 will contain two VNP devices, one tank and one pod device (with accompanying replacement battery), a charger and wall adaptor, one pack of replacement coils (5 piece/pack), and nicotine e-liquids to last four weeks (see Table 1). Participants will be provided with detailed instructions on how to use the VNP products.

Table 1: VNP Contents for Pack 1

**Pack 1 Contents: VNP devices and 4 weeks supply of nicotine e-liquids**			
**Devices:**			
Prefilled Pod device and replacement battery			
Tank device and box of replacement coils (5 coils)			
Wall adaptor and charger			
**Liquids:**		Pods	Tank E-liquid
Mixed Flavours	Tobacco	4 packs (8 pods + 1*)	1 x 30mL bottle
	Menthol	5 packs (10 pods)	1 x 30mL bottle
	Fruit	5 packs (10 pods)	1 x 30mL bottle

* Pod devices include a single pod already in the device

Pack 2 contains four weeks supply of nicotine e-liquids (see Table 2). Participants choose their preferred flavours and e-liquid based on device preference. If participant elects to continue using the tank device, an additional pack of replacement coils (5 piece/pack) will be provided in Pack 2. The strength of the nicotine e-liquid remains the same as Pack 1.

Table 2: VNP Contents for Pack 2

**Pack 2 Contents: 4 weeks supply of nicotine e-liquids**				
		Pods only	Tank e-liquid only	Pods and Tank e-liquid
Mixed flavours preference	Tobacco	5 packs (10 pods)	1 x 30mL bottle	2 packs (4 pods) + 1 x 30mL bottle
	Menthol	4 packs (8 pods)	1 x 30mL bottle	2 packs (4 pods) + 1 x 30mL bottle
	Mango	5 packs (10 pods)	1 x 30mL bottle	3 packs (6 pods) + 1 x 30mL bottle
Single flavour preference	Tobacco	14 packs (28 pods)	3 x 30mL bottles	7 packs (14 pods) + 2 x 30mL bottles
	Menthol	14 packs (28 pods)	3 x 30mL bottles	7 packs (14 pods) + 2 x 30mL bottles
	Berry	14 packs (28 pods)	3 x 30mL bottles	7 packs (14 pods) + 2 x 30mL bottles

Comparator Group

Participants in the NRT arm receive up to eight weeks supply of 4mg nicotine gum or lozenges (mint flavour) in two deliveries of four weeks supply (see Table 3).

Table 3: NRT Contents for Packs 1 and 2

NRT type	Product Specification	Quantity	
		Pack 1<br>4-week supply	Pack 2<br>4-week supply
Gum	Nicotinell 4mg<br>(96p box)	5 boxes (n=480 pieces)	4 boxes (n=384 pieces)
Lozenge	Nicotinell 4mg<br>(144p box)	3 boxes (n=432 pieces)	3 boxes (n=432 pieces)

NRT products are TGA approved, registered on the Australian Register of Therapeutic Goods and available over the counter in Australia. All over the counter medications sent to participants will be accompanied by the appropriate consumer medicine information leaflet.

Text Message Quit Support

The text message support (TMS) program will be provided for a period of five weeks (35 days) and will commence 24-48 hours after baseline interview completion and randomization. The TMS program comprises a total 112 text messages, ranging in frequency from two to five texts per day dependent on which week participants are up to in the TMS program. Higher frequency of text messaging will occur in week two, which coincides with participants designated quit date (day eight post-randomisation). Participants can opt-out of the program at any time by replying STOP to any text message, or by calling or emailing the research team. Instructions on opting out by text will be included in one text every week, stating 'Reply STOP to opt-out'.

The text messages will include text, emojis, and hyperlinks to web-based videos, quit support websites and graphics interchange format (GIF) images, to promote engagement with the program. The TMS program will also incorporate a 'Quit Buddy' persona named Lou, who will be

introduced on the first day of the program e.g. *'My name is Lou and I will be your quit buddy for the next 5 weeks. Let's help you quit, one day at a time!'*. Text messages have a character limit of 160 per text message, excluding any hyperlinks.
